# A fixed-target platform for serial femtosecond crystallography in a hydrated environment

**DOI:** 10.1107/S2052252519014003

**Published:** 2020-01-01

**Authors:** M. L. Shelby, D. Gilbile, T. D. Grant, C. Seuring, B. W. Segelke, W. He, A. C. Evans, T. Pakendorf, P. Fischer, M. S. Hunter, A. Batyuk, M. Barthelmess, A. Meents, M. A. Coleman, T. L. Kuhl, M. Frank

**Affiliations:** a Lawrence Livermore National Laboratory, Livermore, CA 94550, USA; b University of California at Davis, California, USA; c Department of Structural Biology, Jacobs School of Medicine and Biomedical Sciences, Hauptman-Woodward Institute, SUNY University at Buffalo, Buffalo, New York, USA; d Center for Free-Electron Laser Science, Deutsches Elektronen-Synchrotron, Hamburg, Germany; e Linac Coherent Light Source, SLAC National Accelerator Laboratory, Menlo Park, California, USA

**Keywords:** serial crystallography, sample delivery, XFELs, in-vacuum studies, fixed-target platforms, graphene, polymers, thin films, microcrystals, sample hydration

## Abstract

For serial femtosecond crystallography at X-ray free-electron lasers, which entails collection of single-pulse diffraction patterns from a constantly refreshed supply of microcrystalline sample, delivery of the sample into the X-ray beam path while maintaining low background remains a technical challenge for some experiments, especially where this methodology is applied to relatively low-ordered samples or those difficult to purify and crystallize in large quantities. This work demonstrates a scheme to encapsulate biological samples using polymer thin films and graphene to maintain sample hydration in vacuum conditions. The encapsulated sample is delivered into the X-ray beam on fixed targets for rapid scanning using the Roadrunner fixed-target system towards a long-term goal of low-background measurements on weakly diffracting samples. As a proof of principle, we used microcrystals of the 24 kDa rapid encystment protein (REP24) to provide a benchmark for polymer/graphene sandwich performance. The REP24 microcrystal unit cell obtained from our sandwiched in-vacuum sample was consistent with previously established unit-cell parameters and with those measured by us without encapsulation in humidified helium, indicating that the platform is robust against evaporative losses. While significant scattering from water was observed because of the sample-deposition method, the polymer/graphene sandwich itself was shown to contribute minimally to background scattering.

## Abstract 2

This work demonstrates the use of polymer thin films and graphene to support and maintain the hydration of protein microcrystals on fixed targets for serial femtosecond crystallography at X-ray free-electron lasers. Rapid encystment protein (REP24) provides a benchmark for this encapsulation approach.

## 1. Introduction   

The advent of hard X-ray free-electron lasers (XFELs), including the Linac Coherent Light Source (LCLS) at SLAC National Accelerator Laboratory in 2009, has opened up exciting new opportunities for structural biology (Spence & Chapman, 2014 ▸; Feld & Frank, 2014 ▸; Schlichting, 2015 ▸; Johansson *et al.*, 2017 ▸). The ultrafast, high-brightness pulses from XFELs allow the collection of nominally damage-free (Neutze *et al.*, 2000 ▸) single-pulse diffraction images from biological micro- and nano-objects at room temperature, including protein microcrystals (Chapman *et al.*, 2011 ▸; Boutet *et al.*, 2012 ▸), individual virus particles (Seibert *et al.*, 2011 ▸; Sun *et al.*, 2018 ▸) or even weaker diffracting nano-objects, such as two-dimensional protein crystals (Pedrini *et al.*, 2014 ▸; Frank *et al.*, 2014 ▸; Casadei *et al.*, 2018 ▸) or protein fibrils (Seuring *et al.*, 2018 ▸). High-resolution three-dimensional protein structures can be derived from a large collection of individual diffraction patterns (typically thousands for a full diffraction dataset) obtained sequentially from individual microcrystals in random orientations that are replenished in-between X-ray pulses (Kirian *et al.*, 2010 ▸; Chavas *et al.*, 2015 ▸; White *et al.*, 2016 ▸). This serial femtosecond crystallography (SFX) approach is becoming an increasingly routine method for macromolecular structure determination (Yun *et al.*, 2019 ▸; O’Sullivan *et al.*, 2018 ▸; Audet *et al.*, 2019 ▸; Dao *et al.*, 2018 ▸; Stauch *et al.*, 2019 ▸) but challenges remain for the application of this approach for more weakly diffracting samples.

A major challenge in biological imaging with XFELs is the requirement to continuously inject sample to the XFEL focus (1) at a rate that matches the pulse repetition rate of the XFEL (120 Hz in the case of LCLS), (2) in vacuum to minimize background scattering from air, especially in the case of small or weakly diffracting objects, and (3) in such a way that sample hydration is maintained to prevent degradation. Various types of continuous sample delivery systems have been developed (Martiel *et al.*, 2019 ▸); liquid-jet injectors that utilize gas dynamic virtual nozzles to inject hydrated microcrystals and lipidic cubic phase (LCP) injectors (Weierstall *et al.*, 2014 ▸; Nogly *et al.*, 2016 ▸) are workhorses for sample injection for SFX. However, high consumption (∼20 µl min^−1^ and several milligrams of protein for a full SFX diffraction dataset for liquid jets, ∼100 µg for LCP jets) and the X-ray background scatter from the water and buffer components are drawbacks. Tape drives mitigate challenges related to sample consumption by synchronizing drop arrival to the X-ray pulse timing (Fuller *et al.*, 2017 ▸; Beyerlein, Dierksmeyer *et al.*, 2017 ▸) but similarly suffer from background contributions from the relatively large aqueous droplet and the tape material that supports the drop. Aerodynamic lens-based aerosol injectors (Bogan *et al.*, 2008 ▸; Hantke *et al.*, 2014 ▸) have very low scattering background for imaging but the probability of an X-ray shot resulting in a measurable diffraction pattern, or hit rate, is low.

An alternative is to introduce the sample via sample supports which are scanned through the X-ray beam such that fresh sample is introduced for each X-ray shot. This fixed-target approach can drastically reduce the amount of sample required for obtaining a full diffraction dataset. Hit rate can be maximized by increasing sample-deposition density. The fixed-target approach also poses several practical experimental challenges including X-ray background scatter from the sample support, relatively slow speed of data acquisition caused by mechanical stepping or scanning of the sample, and the need to maintain sample hydration when exposed to vacuum.

Various fixed-target approaches for biological imaging at XFELs and at synchrotrons, where serial crystallography approaches with similar challenges are increasingly being implemented, have been investigated over the last decade to address these challenges. A number of sample supports have been developed for serial fixed-target femtosecond crystallography (FT-SFX) and serial synchrotron crystallography including microgrids based on silicon (Zarrine-Afsar *et al.*, 2012 ▸) or polymers (Feld *et al.*, 2015 ▸; Baxter *et al.*, 2016 ▸), silicon or polymer chips with silicon nitride membranes (Hunter *et al.*, 2014 ▸; Frank *et al.*, 2014 ▸; Murray *et al.*, 2015 ▸), and micropatterned silicon chips (Roedig *et al.*, 2015 ▸). Various schemes for mitigating sample dehydration have been employed to maintain a functional species at room temperature including: enclosing microgrids with polymer films (Mueller *et al.*, 2015 ▸; Owen *et al.*, 2017 ▸; Oghbaey *et al.*, 2016 ▸; Ebrahim *et al.*, 2019 ▸; Schulz *et al.*, 2018 ▸; Sherrell *et al.*, 2015 ▸); surrounding microcrystal samples with protective oil, such as Paratone-N (Hunter *et al.*, 2014 ▸) or grease (Sugahara *et al.*, 2015 ▸); embedding 2D crystal samples in sugar (Frank *et al.*, 2014 ▸); or providing a humidified air or helium atmosphere (Roedig *et al.*, 2015 ▸, 2016 ▸, 2017 ▸). While early FT-SFX experiments at LCLS and other XFELs were performed at fairly low scanning speeds (∼10 Hz), the Roadrunner fast-scanning stage system that was developed by the Centre for Free-Electron Laser Science utilizes fast stages that are synchronized with the X-ray pulse repetition rate such that X-ray shots are spatially aligned with the micropores of a micropatterned silicon chip (Roedig *et al.*, 2017 ▸). The sample can be supported in humidified helium with adjustable relative humidity, allowing for bare crystals to be measured on the chip (Roedig *et al.*, 2015 ▸). Roadrunner has also been implemented in ultrahigh vacuum but without adaptation of the supporting chip to mitigate sample dehydration.

To utilize this fast-scanning platform for small crystals, nano-objects and ordered films, compatible strategies to provide a continuous supporting substrate with minimal X-ray scattering background must be developed. In recent years, single-layer graphene, an ultrathin material with excellent mechanical, thermal and barrier properties (Geim & Novoselov, 2010 ▸; Novoselov *et al.*, 2012 ▸), has been used at synchrotron sources for mounting protein crystals in a cryoloop to minimize background and prevent sample dehydration (Wierman *et al.*, 2013 ▸), as a water-barrier film over windows in graphene-based microfluidics (Sui *et al.*, 2016 ▸), and as a low-background support material to align amyloid fibrils (Seuring *et al.*, 2018 ▸). Given its ultrathin nature, stand-alone graphene is difficult to handle and produce without cracks resulting from the etching steps (Li *et al.*, 2009 ▸; Liang *et al.*, 2011 ▸; Borin Barin *et al.*, 2015 ▸). These cracks can severely affect the barrier properties of graphene for in-vacuum studies.

Therefore, our strategy towards background minimization for weakly diffracting samples was to explore the use of large-area few-layer graphene (FLG) in conjunction with polymer thin films, which would impart mechanical robustness, flexibility and allow for easy handling while minimally adding to the X-ray background scatter. We used these hybrid films as enclosing layers to maintain sample hydration for room-temperature studies in the Coherent X-ray Imaging (CXI) end station (Liang *et al.*, 2015 ▸) vacuum environment with micropatterned fabricated Si substrates compatible with a rapid-scanning approach utilizing Roadrunner. A crystal slurry is deposited and spread by capillary force between these enclosing films, limiting physical stress on the crystals. SFX experiments were performed in both the vacuum environment of CXI and the humidified environment at the Macromolecular Femtosecond Crystallography (MFX) end station (Sierra *et al.*, 2019 ▸) without encapsulation. Our initial studies focus on 24 kDa rapid encystment protein [REP24; PDB code 4p5p (Segelke *et al.*, unpublished work)] to provide a benchmark for polymer/graphene sandwich performance. By performing a comparative study of REP24 diffraction encapsulated in-vacuum and without encapsulation in humidified helium, we determine that the device is robust against evaporative losses.

## 2. Materials and methods   

Serial diffraction from batch-grown microcrystals of REP24 was measured on pore-patterned Si chips both at (1) CXI in vacuum with the crystals encapsulated in polymer/graphene hybrid films and at (2) MFX in a humidified environment on a bare Si chip (without encapsulation or sandwiching) to benchmark the performance of the sandwich towards preventing dehydration. The following descriptions of the protein crystallization, the chip design and the performance of the SFX experiments pertain to experiments in both conditions unless otherwise noted.

### 2.1. Preparation of graphene–polymer thin films   

**Figure 1:**
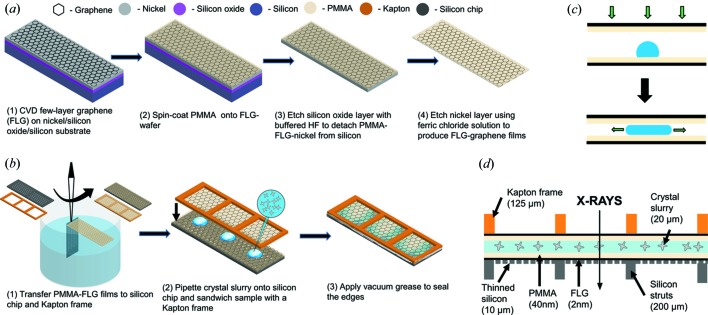
Schematic for (*a*) preparation of PMMA-FLG thin films and (*b*) transfer to substrates, sample deposition and final device assembly. (*c*) Spreading of sample droplet over chip area by capillary action and (*d*) a side-view cross-section of the assembled device not to scale.

Chemical vapor deposited FLG on nickel grown on a 4 inch silicon wafer with a 300 nm silicon oxide layer was purchased from Graphene Supermarket (Calverton, NY, USA) and cut to desired dimensions as required. The deposition process on nickel results in a continuous surface consisting of 3–10 µm sized patches of 1–7 monolayer thickness [Fig. 1 ▸(*a*)]. The wafers were rinsed with a 2 vol.% Hellmanex III and MilliQ water (18.2 MΩ) thoroughly, and dried with high purity nitro­gen prior to use, to remove any silicon particles generated during cutting and handling.

PMMA-assisted transfer of graphene to substrates has been well established in literature (Li *et al.*, 2009 ▸; Liang *et al.*, 2011 ▸; Borin Barin *et al.*, 2015 ▸). 4 wt% poly(methyl methacrylate) (PMMA) in anisole with a molecular weight 950 000 g mol^−1^ (950PMMA A4) was purchased from MicroChem (Westborough, MA, USA). The solution was diluted to 0.8 wt% and spin-coated onto the FLG wafer at 2500 rev min^−1^ for 90 s, followed by annealing at 80°C for 15 min to form a polymer film ∼40 nm thick as measured by profilometry (Dektak 150).

The PMMA-coated FLG wafer was subsequently immersed in buffered oxide etchant (Transene Buffer HF Improved) for 30–60 min to etch the silicon oxide layer, thereby detaching PMMA/FLG/nickel film from the silicon wafer. The nickel layer was etched by transferring this film to the surface of a ferric chloride bath (Transene CE100) diluted 1:9 in MilliQ water (18.2 MΩ) to allow for slow etching, followed by three rinse cycles in MilliQ water (20 min each) to remove any residual etchant. Fig. 1 ▸(*a*) shows the schematic for preparation of PMMA-FLG films. Additional information on graphene-film characterization is presented in the Supporting information (Figs. S1 and S2).

### 2.2. Preparation of REP24 microcrystals   

REP24 [24 kDa; PDB code 4p5p (Segelke *et al.*, unpublished work)], a putative virulence factor protein from the intracellular pathogen *Francisella tularensis*, was expressed recombinantly and purified as described previously (Feld *et al.*, 2014 ▸; Hunter *et al.*, 2014 ▸). Batch crystallization conditions were utilized for the production of the REP24 crystals in bulk. REP24 crystals were grown by mixing a 14.4 mg ml^−1^ REP24 sample (in 50 m*M* NaCl and 10 m*M* HEPES pH 7.5) with a precipitation solution containing 54%(*v*/*v*) PEG-MME 750 and 100 m*M* Na-acetate pH 4.5 in a 1:1 ratio for final conditions of 7.2 mg ml^−1^ REP24, 27%(*v*/*v*) PEG-MME 750, 50 m*M* Na-acetate pH 4.5 in 25 m*M* NaCl, and 5 m*M* HEPES pH 7.5. The crystals of REP24 used in the experiment were between 15 and 20 µm in length and had the appearance of two square-based pyramids connected at the peak, with a maximum thickness of 10 µm. Crystal concentrations were estimated at 2.2 × 10^6^ crystals ml^−1^ based on counts done via optical microscopy of a fixed volume.

### 2.3. Chip design and sample assembly   

Micro-patterned single crystalline silicon chips were commercially manufactured by Finnlitho (Joensuu, Finland), requiring ∼30 day turnover time to take advantage of micro-precise patterning capabilities, which were compatible with the Roadrunner fast-scanning system and are available directly from the manufacturer. All chips used follow the design principles of second-generation Roadrunner chips described previously (Lieske *et al.*, 2019 ▸). Each chip (32.7 × 12 mm) comprised a 200 µm thick frame with an 18 × 5 array of 1.5 × 1.5 mm rectangular areas of Si (windows) thinned to 10 µm and supported by 100 µm-wide struts in between the windows. These windows were patterned with a hexagonal dense pattern of 15 µm pores spaced 50 µm apart, yielding >100 000 pores that could hold crystal samples (Fig. S3). Chips with a slightly different window configuration (6 × 2 array, 7.5 × 4.3 mm) and pores spaced 100 µm apart were used in MFX measurements. Because of the nature of the manufacturing process, the chips have a flat polished side and a structured side that features the recessed areas of the membrane with holes and supporting struts. Chips were screened for breakage or surface damage, sonicated in acetone and iso­propanol for ten minutes each, and dried with ultrapure nitro­gen gas prior to film attachment to the polished side. Kapton frames (Dupont Kapton 500HN, 125 µm) of dimensions 32.7 × 12 mm with large (10 × 10 mm) holes were prepared using a plotting cutter (Cricut Explore Air 2).

#### 2.3.1. Sandwiched sample assembly for CXI   

The chips or Kapton frame were lowered into the water under the PMMA-FLG film floating at the air–water interface and its edge was carefully aligned with the floating film, touched and slowly raised at a 60–90° angle to allow water to recede from under the film as shown in Fig. 1 ▸. The PMMA-FLG coated substrates were allowed to air dry for 15 min followed by annealing at 80°C for 15 min to evaporate any residual water. A PMMA-FLG coated Finnlitho chip is shown in Fig. S3(*b*).

The resulting PMMA-FLG coated chips (composing the bottom layer of a sample sandwich) were mounted film side up to custom-fabricated aluminium frames bearing magnetic mounts for loading onto the goniometer of the fast-scanning Roadrunner systems used for the experiments at the LCLS (Roedig *et al.*, 2016 ▸, 2017 ▸). Alignment pins in the aluminium frames allowed for precise alignment of the chips as they were attached to the aluminium frames with nail polish. Approximately 20 µl of the microcrystal slurry was carefully pipetted onto the chip and a PMMA-FLG coated Kapton frame was then carefully aligned with the chip and placed film side down on top of the microcrystal solution. The solution then spread by capillary action to cover a large area of the chip. A schematic of this spreading action is shown in Fig. 1 ▸(*c*). The edges of the chip and film sandwich were sealed by application of a thin layer of vacuum grease to prevent dehydration. A cross-section of this assembly is shown in Fig. 1 ▸(*d*). Before the beamtime, the sandwich assembly (using silicon chips with larger 50 µm pores) was tested for vacuum stability using an in-house vacuum chamber. The crystals appeared intact after 30 min of vacuum exposure, as seen in Fig. S4(*a*), and in no case did we observe delamination of the assembly. Crystals have no apparent preferred orientation when deposited [Fig. S4(*b*)].

#### 2.3.2. Sample preparation for MFX   

For humidified environment experiments at MFX, bare Finnlitho silicon chips were loaded with 50 µl of freshly crystallized REP24 microcrystal suspension within a specialized humidified chamber by pipetting and spreading the crystal slurry onto the flat side of the chip, and wicking away excess mother liquor from the opposite side to aid in drawing microcrystals into the chip pores (Roedig *et al.*, 2016 ▸, 2015 ▸). The loaded chips were then immediately transferred to the Roadrunner sample chamber, which was constantly flushed with humidified helium (near 100% humidity).

### 2.4. Serial femtosecond crystallography data collection   

SFX experiments took place during two brief six hour Protein Crystal Screening beamtimes. They were conducted at the LCLS both in humidified atmosphere and in vacuum using the humidified helium environment Roadrunner III system at the MFX end station and the newly developed vacuum-compatible Roadrunner IV system in the 0.1 µm *in vacuo* sample environment of the CXI end station, respectively. Roadrunner III and IV systems share main design elements with previous versions of the Roadrunner system which have been demonstrated at the LCLS and elsewhere in the past and their capabilities have been described (Beyerlein, Dierksmeyer *et al.*, 2017 ▸; Roedig *et al.*, 2017 ▸, 2016 ▸, 2015 ▸). These include high-precision stepper motor-driven *x* *y* *z* translation stages, a brushless motor linear stage for high speed scanning of chips oriented in the horizontal direction and a high-precision goniometer. Development of Roadrunner III and IV is not the focus of this work and will be described in forthcoming publications.

The procedure for sample mounting and data collection is similar for both systems. After mounting, chips were scanned through the X-ray focus row-by-row, where the *x* axis was the fast-scanning axis. Scanning speed was precisely controlled such that the arrival of each X-ray pulse at the 120 Hz repetition rate of the LCLS was coincident with the spatial alignment of a micropore on the chip. The scanning was facilitated by a chip-geometry definition file of each chip design that was pre-loaded into the Roadrunner data-collection software before the experiments, allowing the software to calculate the necessary acceleration and velocities to synchronize the arrival of a chip micropore with the LCLS pulse. A high-resolution in-line viewing microscope was used to view and align the samples.

For in-vacuum experiments, samples were loaded and exposed to vacuum during the initial 20 min pumpdown of the sample chamber and subsequent 30 min runtime of the chip (including ∼20 min data-collection time and ∼10 min deadtime). For experiments in humidified helium, the chips were immediately transferred to the Roadrunner III sample chamber flushed with humidified helium (99 to 100% humidity) after loading. Humidity at the sample was monitored throughout the course of the experiment to ensure >99% humidity.

SFX experiments were conducted at X-ray energies of 7.5 keV and 9.5 keV with a beam size at the sample of 120 × 170 nm full width at half-maximum (FWHM) and 3 × 3 µm FWHM for experiments at CXI (Schropp *et al.*, 2013 ▸) and MFX (humidified atmosphere), respectively. REP24 samples were measured with between 1 and 10% beam transmission and not the full X-ray flux of 4.5 mJ pulse^−1^ because of (1) the saturation of detector pixels within Bragg spots at higher flux and (2) concerns regarding damage to the chip resulting from the lower-intensity wings of the X-ray beam around the central focus spot (∼1% of the total intensity). All chips were assessed for damage immediately following measurement with optical microscopy. The nominal pulse duration was 40 fs for both experiments. During each sample scan, diffraction images were recorded on a shot-by-shot basis at the full 120 Hz repetition rate of the LCLS by the Cornell-SLAC Pixel Array Detector (CSPAD; Blaj *et al.*, 2015 ▸). As data were collected, X-ray images were analyzed to estimate the hit rate using *OnDA* for immediate feedback (Mariani *et al.*, 2016 ▸). Images from all X-ray shots were analyzed offline using *Cheetah* (Barty *et al.*, 2014 ▸) to find crystal hits with the following parameters: minimum peak intensity threshold of 200 ADU (analogue-to-digital units), minimum signal-to-noise ratio of 6, minimum number of pixels per peak of 2 and minimum number of peaks per hit of 10. *CrystFEL* (White *et al.*, 2012 ▸) was used for indexing patterns and to estimate resolution using the *MOSFLM* indexing algorithm (Battye *et al.*, 2011 ▸).

## 3. Results and discussion   

### 3.1. SFX experiments   

**Figure 2:**
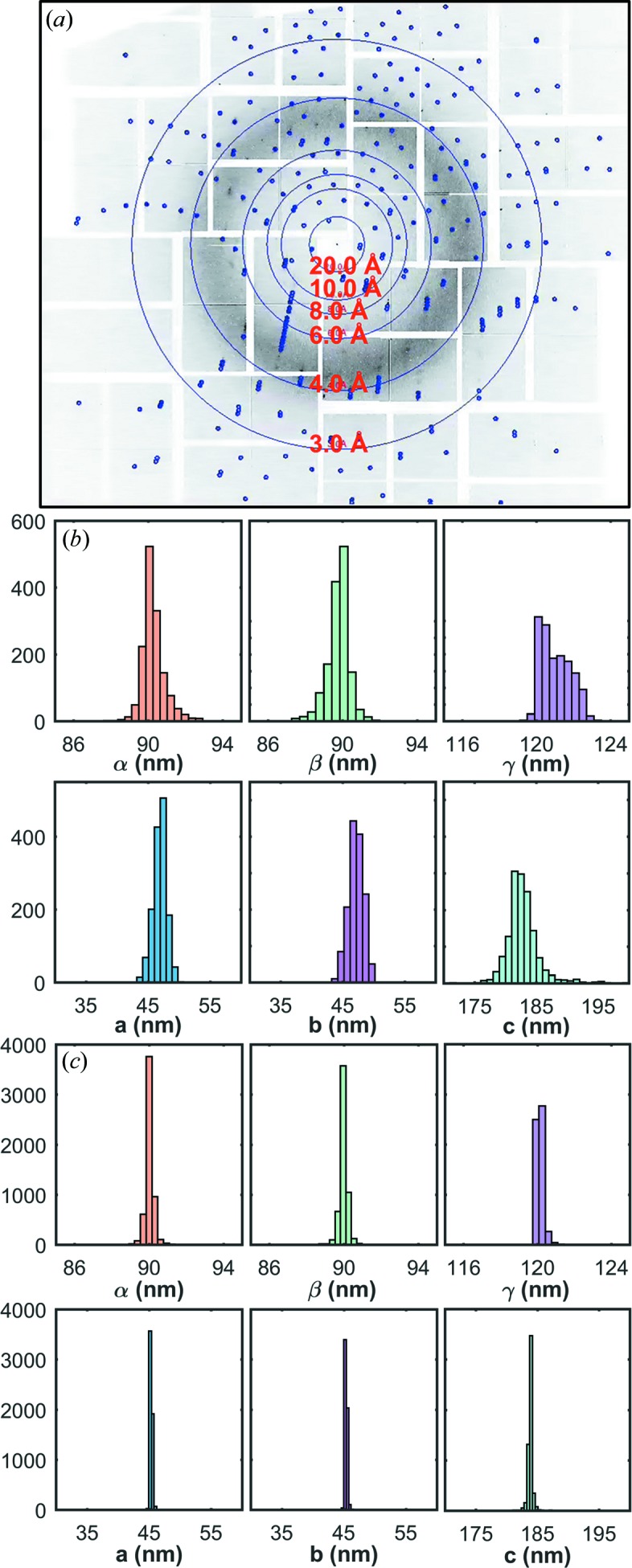
(*a*) REP24 microcrystals diffract up to ∼2.2 Å. No degradation of resolution was observed as the exposure time to vacuum increased. (*b*) Histograms of unit-cell parameters from ∼1400 indexed patterns in vacuum and (*c*) ∼5500 indexed patterns in humidified helium.

In the humidified helium environment at MFX, CSPAD images from ∼21 000 shots were recorded with ∼8000 containing hits (38% hit rate) of REP24 microcrystals on the bare chip, of which 5500 (69%) were successfully indexed. The indexed unit-cell dimensions were *a* = 45.3 ± 0.1 Å, *b* = 45.3 ± 0.1 Å, *c* = 183.9 ± 0.2 Å, α = 90.0 ± 0.1°, β = 90.0 ± 0.1° and γ = 120.1 ± 0.1° (Fig. 2 ▸). The maximum per-frame resolution was 1.9 Å while the median resolution was 2.3 Å. During SFX experiments at CXI in vacuum, ∼60 000 shots were recorded, ∼4800 of which contained measurable diffraction data from microcrystals encapsulated in the sandwich and were determined to be hits (8% hit rate, see Section 3.3 for a comparison of the hit rate). Of these hits ∼1400 (30%) were successfully indexed and exhibited unit-cell constants of *a* = 47.0 ± 1.0 Å, *b* = 47.2 ± 1.2 Å, *c* = 183.5 ± 1.6 Å, α = 90.2 ± 0.4°, β = 89.9 ± 0.4° and γ = 120.9 ± 1.0° (Fig. 2 ▸).

The observed unit cell in both cases is consistent within the error of the respective measurements with the unit cell determined for larger REP24 crystals by synchrotron experiments at cryogenic temperatures (PDB code 4p5p; Segelke *et al.*, unpublished work), with unit-cell parameters of *a* = 44.4 Å, *b* = 44.4 Å, *c* = 183.5 Å, α = 90.0°, β = 90.0° and γ = 120.0°. Previous room-temperature measurements of REP24 using fixed-target SFX also yielded a comparable unit cell as synchrotron single-crystal measurements: *a* = *b* = 44.4 Å, *c* = 183.5 Å, α = β = 90°, and γ = 120°, indicating that cryogenic cooling does not significantly impact the unit cell. They utilized similar micro-crystallization conditions but adopted a very different approach to preventing dehydration in which the microcrystals were suspended in Paratone-N and then spread onto a silicon nitride support, which is not applicable to weakly diffracting samples (Hunter *et al.*, 2014 ▸). The slightly larger average unit cell within the sandwich may indicate that the enclosure of the microcrystals inhibits any loss of hydration caused by removal from the mother liquor for mounting or suspension in Paratone-N, as was performed for the MFX experiment and previous measurements of REP24.

Sandwich encapsulated crystals diffracted to a maximum resolution of 2.2 Å, comparable with previous SFX experiments, and a median resolution of 3.5 Å (Fig. 2 ▸). Neither the diffraction-limit distribution nor the mean diffraction limit changed significantly during the measurement time. The unit-cell volume distribution was also constant throughout the experiment (Fig. S5), indicating that the PMMA–FLG sandwich afforded a high degree of protection against dehydration to the microcrystals during the experiment and prevented degradation of diffraction quality. Insufficient data were collected for full structure determination for in-vacuum experiments as determined by the *R*_split_ figure of merit (75.0% for the entire dataset; White *et al.*, 2012 ▸). Statistics for the reflections collected according to resolution shell are tabulated in Table 1 ▸.

It is notable that the maximum and mean resolution of REP24 measured in humidified helium showed some improvement over those measured in-vacuum within the sandwich, primarily in that the distribution of maximum frame resolution was much wider at CXI, with a standard deviation of 1.1 Å in vacuum *versus* 0.3 Å in humidified helium. There is also a much larger variance in the unit-cell dimensions for the CXI dataset, which is evident in the histograms of the indexing solutions (Fig. 2 ▸), although this is partially caused by the lower number of indexed hits during the in-vacuum experiment. The source of this heterogeneity and whether it originates from (1) interactions between the microcrystals and the graphene or polymer surface, (2) partial drying of the sample, or (3) crystallization of a more non-uniform crystal slurry caused by differences in purified protein batch or crystallization conditions is not currently clear. One would expect drying to result in a time-dependent shrinkage or a general decrease of the unit-cell volume compared with the experiment in humidified helium, which does not appear to be the case. While interactions between the microcrystals and the graphene or polymer surface are not ruled out, one might expect this to manifest in more of a bimodal distribution with a population that strongly interacts with the surface and one that does not. Batch crystallization during the CXI beamtime resulted in a more varied distribution of crystal sizes according to optical images taken before measurement, which provides some indication that (3) is the most likely explanation of this phenomena.

### 3.2. Polymer–graphene sandwich performance   

**Figure 3:**
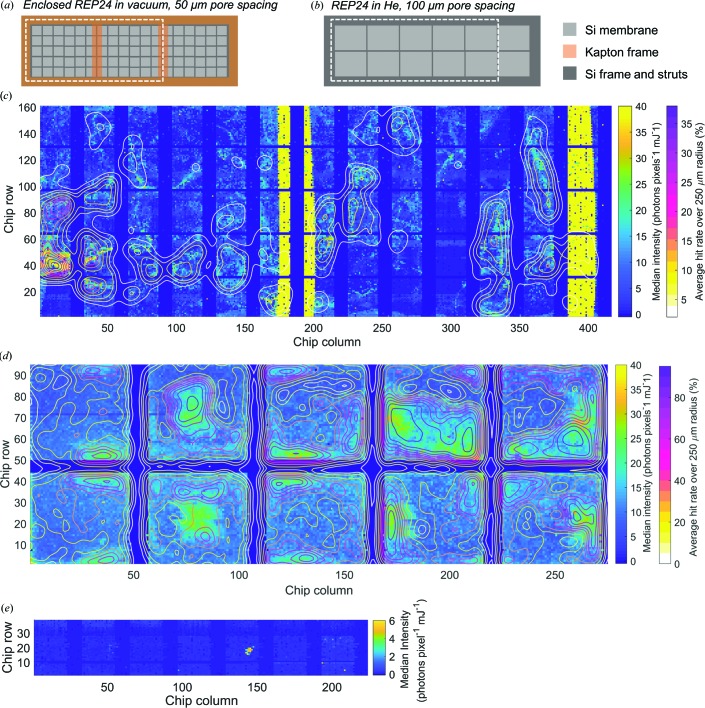
Areas of chips measured for REP24 at (*a*) in-vacuum at CXI and (*b*) in humidified He at MFX, represented by the median-intensity heat maps below. Heat maps of median scattered intensity overlaid with contour plots of hit rate averaged spatially over a 250 µm radius for (*c*) the enclosed REP24 sample at CXI, (*d*) the REP24 sample at MFX and (*e*) the thin-film/PMMA-FLG sample at CXI.

The relatively low hit rate for the in-vacuum experiment compared with the humidified helium experiment (∼8% *versus* 38%) can be explained by the difference in crystal deposition methods for each experiment. The sample was unsupported in humidified helium and excess liquid was removed after deposition by wicking on the opposite side of the chip, eliciting a focusing effect where crystals are drawn into pores by the wicking process. The sandwiching method employed in vacuum relies on capillary action to spread the solution applied to the first PMMA–FLG film when the enclosing PMMA–FLG layer is applied. While the hit rate reported for the in-vacuum experiment was relatively low compared with both the MFX experiment and the ideal hit rate (for randomly distributed crystals and Poissonian statistics, the maximum single-crystal hit rate of ∼37% is achieved at a total hit rate of ∼63%), higher hit rates can easily be achieved by concentrating the microcrystal slurry by centrifugation before application to the chip to be sandwiched. The crystal density across the chip is not uniform because of this process of spreading and this was observed as an unevenness in the hit rate as the chips were scanned in these studies [Fig. 3 ▸(*c*)].

Though preparation of the PMMA–FLG hybrid films was somewhat labor intensive because of multiple etching and washing steps, we found the prepared films could be transported to the beamline on mica and transferred to the silicon chip and Kapton frame components shortly before beamtime. The actual sandwich assembly was done on the fly throughout the beamtime, and sandwiched microcrystal slurries were stable for several hours at room temperature and atmospheric pressure while they awaited measurement. The thinness of the enclosed water layer inhibited any redistribution of the crystals during this waiting period and no gravity-related pooling of crystals to the bottom of the chip was observed during measurement.

Intact microcrystals were observed in areas of the chip between pores (and therefore not exposed to X-rays) after measurement, suggesting that hydration is locally maintained despite physical damage to the chip and the membrane substrates. Currently, damage to both the PMMA–FLG films and the Si membrane itself as a result of beam exposure makes the assembly a one-time-use under these measurement conditions. While in the case of REP24 permissible X-ray flux on the chip was limited to avoid detector saturation within Bragg spots, the Si chip itself imposes a limit to incident flux. While the nominal beam sizes (FWHM) for both experiments are well within the pore sizes used, there are spatially broad wings that are still sufficiently intense to cause significant damage to the chips themselves. This becomes problematic if this damage either causes melting of the Si substrate and thus significant amorphous Si scatter signal, or if this damage causes cracks that propagate down a row of pores resulting in physical deformation of the chip. While strong single-crystal Si Bragg reflections are at higher angles than typically observed for macromolecular crystals, rotation of the Si membrane due to this physical damage may result in these potentially damaging reflections appearing on the detector. This bolsters the case for development of amorphous polymer chip materials both to prevent unintended exposure of the detector to strong reflections resulting from damage and to reduce manufacturing costs.

The unit cell of REP24 found by analysis of diffraction data derived from in-vacuum and humidified atmosphere experiments was in good agreement and indicates that the presented method of sample enclosure with graphene and polymer films is robust against evaporative losses. To our knowledge, other methods utilizing polymer films and/or graphene as supporting or enclosing substrates for XFEL or synchrotron studies either used much thicker polymer films (micrometres thick) alone (Doak *et al.*, 2018 ▸; Mueller *et al.*, 2015 ▸; Owen *et al.*, 2017 ▸; Oghbaey *et al.*, 2016 ▸; Sherrell *et al.*, 2015 ▸; Ebrahim *et al.*, 2019 ▸) to support graphene (Sui *et al.*, 2018 ▸, 2016 ▸), or did not provide continuous coverage over the entire area of the chip because of the difficulties with handling large areas of unsupported graphene (Seuring *et al.*, 2018 ▸). Thicker films do provide a more robust barrier to evaporation but require larger microcrystals to compensate for increased X-ray background scatter. For relatively low-order and poorly diffracting objects, it will be critical to minimize the background contribution of the sample enclosure while maintaining sample hydration and crystal integrity. Therefore, the sandwiching approach presented here is expected to be beneficial compared with some of the other approaches.

### 3.3. Origin of contributions to background scattering   

In an effort to understand the contributions to the background of the enclosed sample, device and sample environment, median scattering intensities were calculated for REP24 within the PMMA-FLG sandwich, a PMMA-FLG sandwich containing a non-crystalline thin film (∼5–10 nm) sample consisting of protein and lipid and deposited onto the chip without water, and REP24 on the bare Si chip in humidified He at MFX. These median intensities were arranged to spatially represent variations in background across the chip. Radial averages of each detector frame for both MFX and CXI experiments were calculated, normalized according to the incident pulse energy and degree of beam attenuation, and converted to photons per pixel by identifying the detector response corresponding to the single photon peak in a histogram of single-pixel detector response for the whole detector. The median photons per pixel was calculated per shot for each scattering profile to exclude sharp peaks resulting from Bragg reflections. This intensity was sorted by time stamp and the chip-row number recorded by the Roadrunner translation software [Figs. 3 ▸(*c*)–3(*e*)].

**Figure 4:**
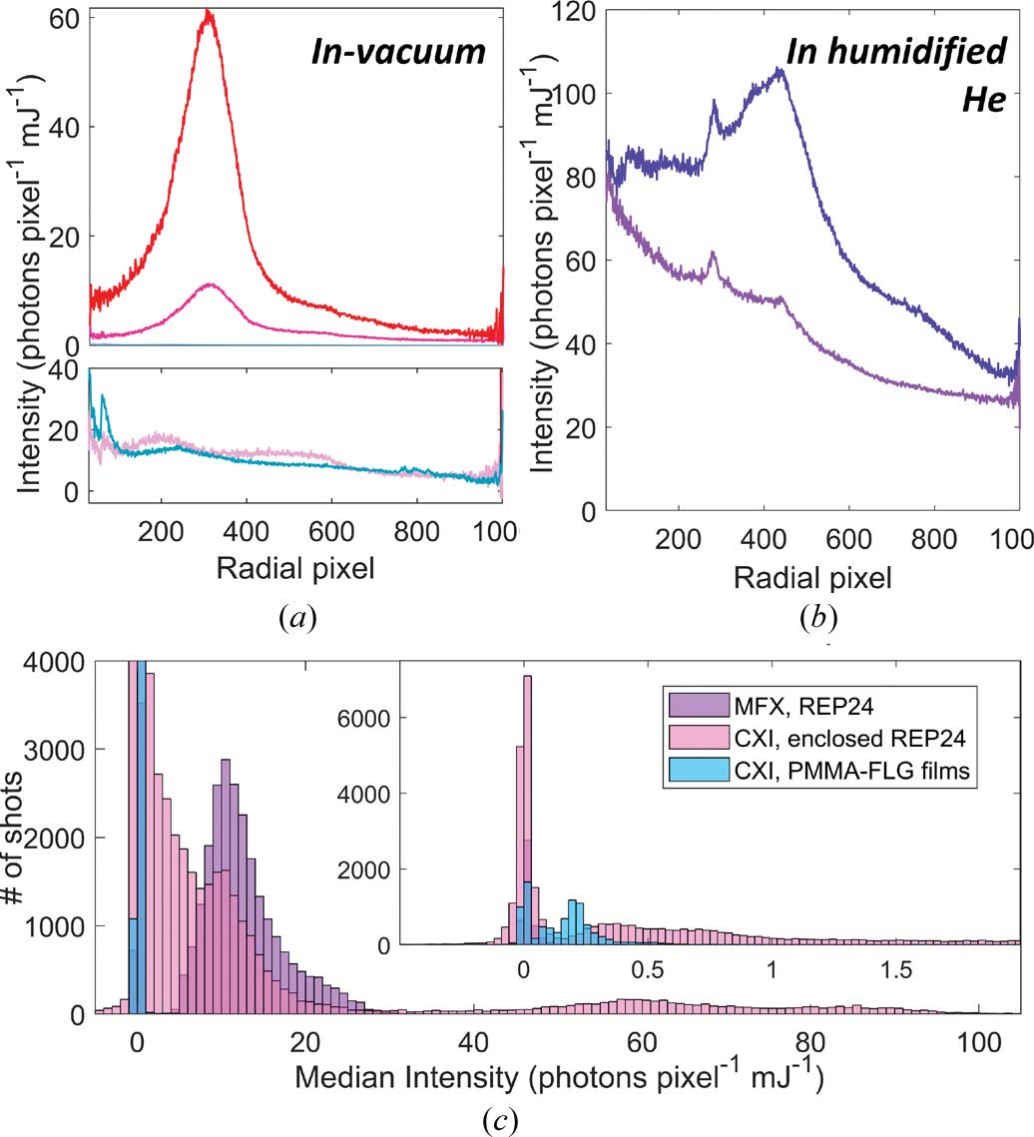
Representative radial scattering profiles for (*a*) high-intensity (red) moderate-intensity (fuchsia) and low-intensity (light pink) frames of enclosed REP24 as well as the PMMA–FLG thin film sample (light blue) in-vacuum at CXI and (*b*) high-intensity (dark purple) and low intensity (light purple) frames of REP24 in humidified He at MFX. (*c*) Histograms of median intensity and (inset) low-intensity populations zoomed in.

In general, the chip with sandwiched REP24 contains three populations: very high intensity shots above 40 photons pixel^−1^, very low intensity shots below 5 photons pixel^−1^, and a population with variable intensity between ∼10 and 35 photons pixel^−1^ [Fig. 3 ▸(*c*)]. The majority of the high-intensity shots correspond to the location of the Kapton frame used to support the second enclosing layer of PMMA–FLG film. The shots of moderate intensity are not uniformly distributed across the rest of the chip but are clustered in regions. Representative radial scattering profiles for shots distributed through these regions [Fig. 4 ▸(*a*)] indicate that the dominant component of this varying signal is a broad peak, which corresponds to liquid water (Hura *et al.*, 2003 ▸). A contour-plot overlay representing hit rate over a 250 µm diameter indicates that the hits are localized in areas with relatively high water background [Fig. 3 ▸(*c*)]. The localization of hits around pockets of thicker water layer is probably caused by the manual method of deposition that relies on the capillary force of the buffer to spread the sample. To optimize sample density and hit rate while minimizing water-film thickness, methods could be imagined that deposit many more concentrated drops over the sample area before encapsulation, redesign the chip to allow controlled wicking for water removal, or employ surface functionalization to fix crystals to the film surface before water removal.

The PPMA-FLG sandwich containing the thin-film sample at CXI is extremely uniform and the vast majority of shots have median intensities of <1 photon pixel^−1^ mJ^−1^ [Fig. 3 ▸(*e*)]. These shots are similar to the low-intensity population of shots for the sandwiched REP24 chip, both in their median intensities and in their radial scattering profiles [Fig. 4 ▸(*a*)]. The median intensities of the chip measured at MFX have a higher floor, and the areas of highest intensity are also clustered in certain regions of the chip that mostly correspond to higher hit rates [Fig. 3 ▸(*d*)]. Scattering profiles of shots both at relatively low intensity and high intensity have a broad sloping background that is probably caused by scattering from the humidified He environment. High-intensity shots also have a broad peak that is probably caused by residual water [Fig. 4 ▸(*b*)]

As a point of comparison to our experimental results, the total number of photons scattered (*N*_scat_) onto the CSPAD for each component of the enclosure, PMMA, FLG and water, was modeled based on the following formula,
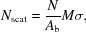
where *N* is the number of incident photons, *A*_b_ is the area of the beam, *M* is the number of atoms or molecules in the beam, and 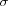 is the atomic or molecular Rayleigh scattering cross-section (from the *xraylib* library; Schoonjans *et al.*, 2011 ▸). Calculated scattered photons are tabulated in Table 2 ▸ for the components relevant to our study and other films used for microcrystal encapsulation. For two PMMA-FLG films consisting of four layers of graphene and 40 nm of PMMA, modeled graphene and PMMA scatter 1.0 × 10^5^ and 1.8 × 10^6^ photons, respectively, per 1 mJ of incident pulse energy at 7.5 keV. A 1 µm film of water scatters 7.8 × 10^6^ photons and we anticipate the water thickness surrounding the crystals to be the same dimension as the crystal size, ∼20 µm, thus the total contribution from water is about 1.6 × 10^8^ photons. This is consistent with our observation that the water signal dominates the background for our encapsulated sample. While more water is removed from the chip at MFX and water scatters less at 9.5 keV (6.6 × 10^6^ photons for 1 µm) the contribution from water vapor for He at 100% humidity at room temperature is likely to be significant (2.6 × 10^8^ photons cm^−1^). By contrast, Mylar films used to sandwich microcrystals in (Doak *et al.*, 2018 ▸), where the water layer is reported to be a similar thickness, would scatter 1.4 × 10^8^ photons per 1 mJ of incident pulse energy at 7.5 keV for two 2.5 µm films.

The very low background intensities across the chip for the thin-film sample and the nature of the varying background for the enclosed sample indicate that the vast majority of the enclosed sample background originates from an encapsulated water layer of varying thickness. The polymer, graphene and Si components of the device together contribute on average <1 photon pixel^−1^ mJ^−1^ to the background, which is ideal for measurement of weakly diffracting objects. While the thickness of the water layer prevented a truly low background measurement in this case, the manual deposition process can be optimized to reduce or remove the excess water enclosed. While the wicking of excess buffer from the bare chip at MFX appears to have reduced the liquid water background, this effect is largely counteracted by the broad background from humidified He. As can be seen from the distribution of shot intensities [Fig. 4 ▸(*c*)], the enclosed sample in vacuum shows most images have low background below 10 photons pixel^−1^ mJ^−1^ with no effective lower limit, whereas the MFX sample in humidified He shows most images contain above 10 photons pixel^−1^ mJ^−1^, with a lower limit of 5 photons pixel^−1^ mJ^−1^. These results demonstrate that the background scattering of the enclosed chip in vacuum can be significantly less compared with the background scattering of the humidified He environment.

## 4. Outlook   

New fast-scanning systems have made fixed-target data acquisition at the 120 Hz repetition rate of the LCLS possible, drastically reducing measurement time for fixed-target SFX experiments and making studies of low-order samples feasible in a relatively short time frame. Recently, even higher scanning rates up to 1 kHz have been demonstrated using a Roadrunner system together with the JUNGFRAU 1M detector at the European Synchrotron Radiation Facility where precise alignment with pores is not a concern (Tolstikova *et al.*, 2019 ▸). While some of the other delivery methods also have the benefit of low sample consumption, for instance LCP and high-viscosity jets, background reduction is critical for both small micro- (or nano-) crystals and samples exhibiting poor diffraction or diffuse features. Diffuse continuous diffraction arising from translational disorder in 3D crystals, especially membrane proteins, has recently been interpreted for phasing and extension of structural resolution (Ayyer *et al.*, 2016 ▸; Chapman *et al.*, 2017 ▸; Morgan *et al.*, 2019 ▸) making a case for background reduction to resolve these features. The reported method enables low-background, high-repetition-rate measurements of such samples while maintaining a near native hydrated environment to make SFX more accessible to new kinds of biological studies.

The next generation of high-repetition-rate XFELs, including the newly opened European XFEL and LCLS-II, currently under construction, will provide potentially much higher rates of data acquisition but also introduce new challenges to sample delivery for SFX. Delivery schemes that minimize downtime (for instance because of injector clogging or fixed-target sample exchange) and provide a modular, well controlled sample environment will be better adapted to take advantage of the faster rate of data acquisition. To this end, we are currently adapting this sample-support-enclosure approach to include low-cost polymer frames that can maintain sample hydration over long periods of time, and substrate functionalization and patterning to control sample deposition and location. This could include patterning to control wetting during deposition or on-chip *in situ* crystallization where nucleation sites are patterned over pores to conserve sample and increase hit rate. Higher-repetition-rate XFELs also provide compelling motivation for future fixed targets to employ a more slot-like window design that requires less accurate spatial synchronization with the X-ray pulse, much like the frameless approach recently demonstrated (Doak *et al.*, 2018 ▸). This also provides more potential sample area and therefore more possible shots per chip. Advances in analysis methodology to interpret data from multiple-crystal hits will further facilitate high sample loading (Beyerlein, White *et al.*, 2017 ▸). Future iterations of chip design would ideally include implementation of microfluidics and/or electrodes for time-resolved experiments using mixing or an applied electric field pulse to trigger interesting biological processes. Substrate patterning could also ensure sample/surface adhesion to facilitate future applications for time-resolved mixing experiments.

## Supplementary Material

Additional characterization of the graphene/polymer films and time invariance of the REP24 unit cell after vacuum exposure. DOI: 10.1107/S2052252519014003/ec5015sup1.pdf

## Figures and Tables

**Table 1:** Statistics for the reflections collected for REP24 in the PMMA-FLG enclosure

Resolution (Å)	Number of reflections observed	Number of possible reflections	Completeness of data (%)	Total measured reflections	Redundancy
7.3	3213	3213	100.0	175780	54.7
3.6	3212	3212	100.0	101608	31.6
3.0	3178	3178	100.0	83537	26.3
2.7	3208	3208	100.0	72609	22.6
2.5	3160	3161	100.0	68412	21.6
2.3	3217	3218	100.0	52385	16.3
2.2	3167	3176	99.7	34518	10.9
2.1	3131	3233	96.9	21996	7.0
2.0	2828	3185	88.8	13011	4.6
1.9	1981	3176	62.4	6464	3.3
Total	30295	31960	0.9	630320	20.8

**Table 2:** Computed scattered photons under measurement conditions for various device components and other potential sources of background scattering

Material	Thickness	Energy (keV)	Total scattered photons at 1 mJ pulse^−1^
Graphene	8 layers	7.5	1.0 × 10^5^
PMMA	80 nm	7.5	1.8 × 10^6^
Water	1 µm	7.5	7.8 × 10^6^
	20 µm	7.5	1.6 × 10^8^
	1 µm	9.5	6.6 × 10^6^
Water vapor at 100% humidity at 20°C in He	1 cm	9.5	2.6 × 10^8^
He at 20°C	1 cm	9.5	7.2 × 10^5^
Mylar	5 µm	7.5	1.4 × 10^8^
