# Supplementary material for: A fixed-target platform for serial femtosecond crystallography in a hydrated environment
Source: IUCrJ. 2020 Jan 1;7(Pt 1):30–41. doi: 10.1107/S2052252519014003 (PMC6949605; doi:10.1107/S2052252519014003)
Supplement: Supplementary file 1 [file m-07-00030-sup1.pdf]

IUCrJ

Volume 75 (2019)

Supporting information for article:

A fixed-target platform for serial femtosecond crystallography in a hydrated environment

M. L. Shelby, D. Gilbile, T. D. Grant, C. Seuring, B. W. Segelke, W. He, A. C. Evans, T. Pakendorf, P. Fischer, M. S. Hunter, A. Batyuk, M. Barthelmess, A. Meents, M. A. Coleman, T. L. Kuhl and M. Frank

Graphene film characterization: Scanning Electron Microscopy using Hitachi S-4100T FE-SEM and Energy Dispersive X-ray Spectroscopy using Oxford INCA Energy EDS were performed on a graphene film transferred to a silicon wafer to characterize film cleanliness, particularly to test for the complete removal of Nickel metal layer from the etching steps. EDX images confirm absence of any residual metal contamination as no peaks corresponding to Nickel are observed. (Fig. S1, S2).

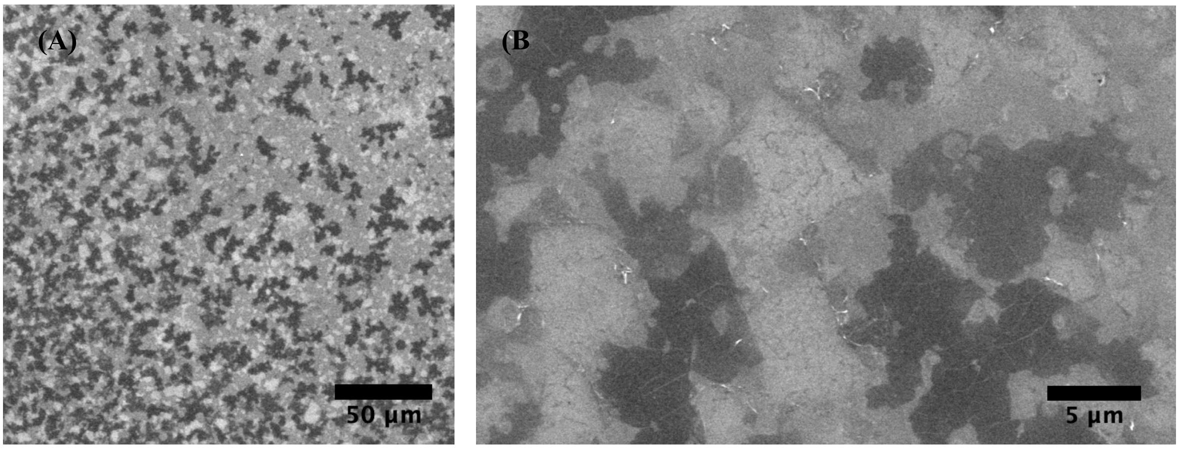

Figure S1 Scanning electron microscopy (SEM) images of few-layer graphene (FLG) transferred to a silicon wafer showing graphene domains with varying thickness at (A) 300X and (B) 3000X magnification.

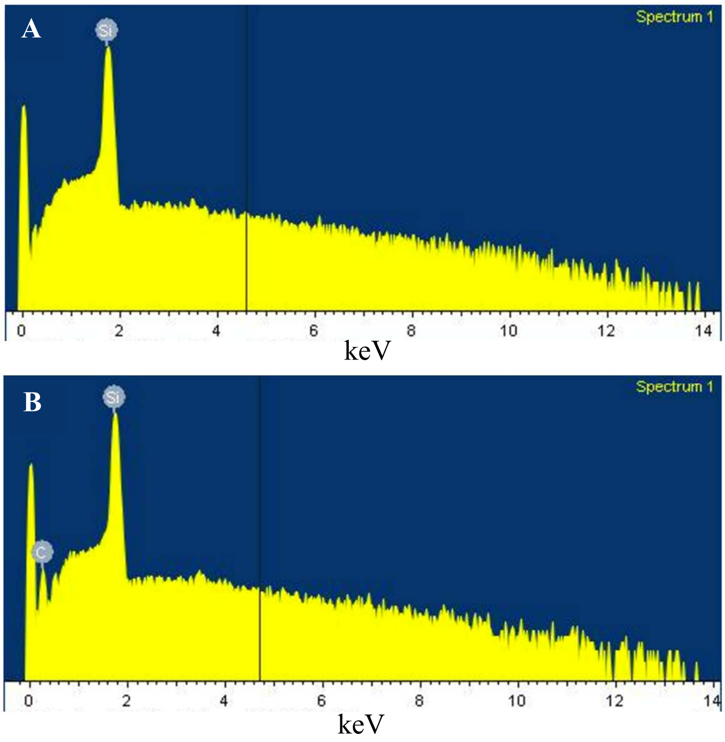

Element	Weight%
Si	100.00
Total	100.00

Element	Weight%
C	7.32
Si	92.68
Total	100.00

Figure S2 Energy dispersive x-ray spectroscopy (EDS) comparison of a (A) bare silicon wafer and (B) few-layer graphene coated silicon wafer confirms the absence of metal contaminants on the graphene surface from the etching steps.

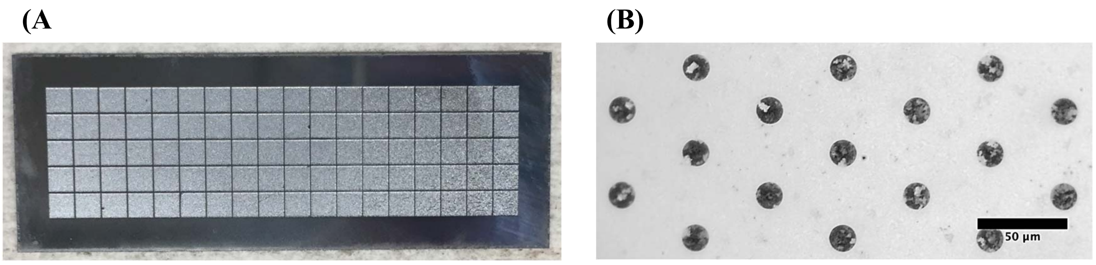

Figure S3 (A) Design of the micropatterned Finnlitho silicon chip with dimensions 32.7 mm x 12 mm. Lighter regions (5 x 18 array) correspond to thinned silicon bearing the hexagonal pore pattern. (B) Optical microscopy image showing the hexagonal pore pattern in a Finnlitho chip covered with a FLG-PMMA film. The graphene films consist of multilayer grains of varying thickness, thicker grains with 6-7 layers of graphene appear as irregular blotches.

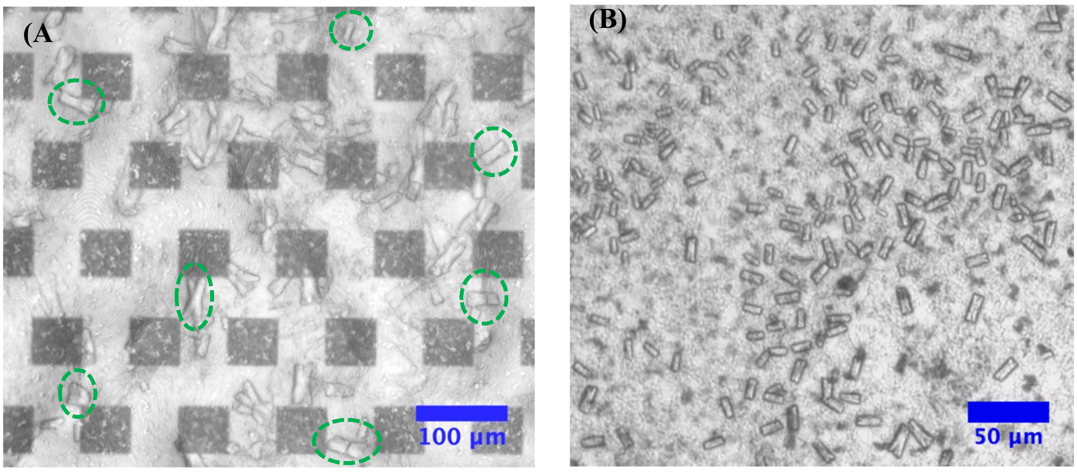

Figure S4 REP24 crystals (A) sandwiched between two few-layer graphene-PMMA hybrid films after 30 minutes in a benchtop vacuum oven at room temperature and (B) deposited on a graphene surface, showing randomly oriented crystal configurations.

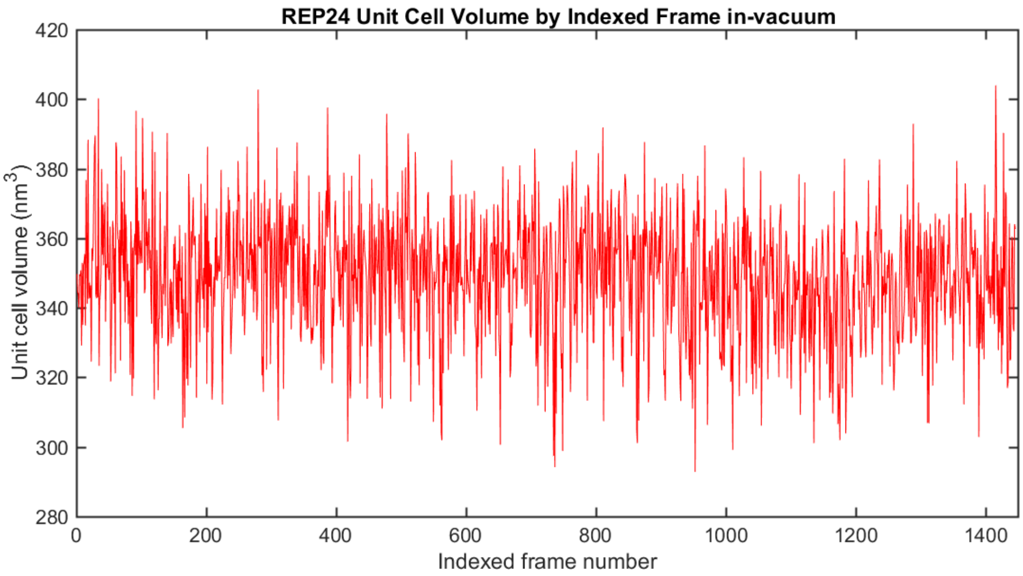

Figure S5 Unit cell volume calculated from unit cell parameters generated by frame-by-frame indexing of REP24 SFX data. These data were collected on REP24 microcrystals encapsulated within the polymer/graphene sandwich and measured in-vacuum over approximately 20 minutes.
